# Parental obesity, health determinants, and cardiometabolic risk according to sleep duration in schoolchildren: analysis through structural equations

**DOI:** 10.1186/s13052-024-01800-z

**Published:** 2024-11-09

**Authors:** Caroline Brand, Vanilson Batista Lemes, Ana Paula Sehn, Cesar Agostinis-Sobrinho, Fernanda Henriquez-Maquehue, Emilio Jofré-Saldía, Paulina Ibacache-Saavedra, Claudio Farias-Valenzuela, Emilio Villa-González, Cézane Priscila Reuter

**Affiliations:** 1 IRyS Group, Physical Education School, Pontificia Universidad Católica de Valparaíso, Valparaíso, Chile; 2 School of Physical Education, Physiotherapy and Dance. Graduate Program in Human Movement Sciences, Federal University of Rio Grande do Sul (UFRGS), Porto Alegre, RS Brazil; 3 Graduate Program in Health Promotion, University of Santa Cruz do Sul (UNISC), Santa Cruz do Sul, RS Brazil; 4 Faculty of Health Science, Klaipeda University, Klaipeda, Lithuania; 5 Facultad de Salud y Ciencias Sociales, Universidad de Las Americas, Sede Providencia, Manuel Montt 948, Santiago, Chile; 6 Exercise and Rehabilitation Sciences Institute, School of Physical Therapy, Faculty of Rehabilitation Sciences, Universidad Andres Bello, Santiago, Chile; 7 Facultad de Ciencias para el Cuidado de la Salud, Universidad San Sebastián, Santiago, Chile; 8 Department of Physical Education and Sports, Faculty of Sport Sciences, Sport and Health University Research Insitute (iMUDS), University of Granada, Granada, Spain; 9 Escuela de Ciencias de la Actividad Física, el Deporte y la Salud, Universidad de Santiago de Chile USACH, Santiago, 9170124 Chile

**Keywords:** Physical fitness, lifestyle, Physical activity, Children, Adolescents

## Abstract

**Background:**

the independent association between parental obesity, sleep and lifestyle habits with cardiometabolic risk factors in children and adolescents has been widely explored in the literature. Our study represents a novel approach to comprehensively investigate a complex model encompassing various aspects associated with cardiometabolic risk in youth. Thus, the aim of the present study was to verify the relationship between parental obesity and cardiometabolic risk in children and adolescents, considering the mediator role of health indicators according to sleep time.

**Methods:**

This cross-sectional study was conducted on 3,973 children and adolescents aged 6 to 17 years attending public and private schools in a city located in Southern Brazil. Sleep duration, lifestyle, physical activity, natural food consumption, and parental obesity were evaluated through self-reported questionnaires. Physical fitness was evaluated according to the protocols of *Projeto Esporte Brasil.* The body fat percentage was evaluated through the measures of tricipital and subscapular folds, and the body mass index was calculated. The clustered metabolic risk score (cMetS) considered the summing z-scores of waist circumference, systolic blood pressure, triglycerides, total cholesterol/high-density lipoprotein cholesterol ratio, and fasting glucose, divided by five. A structural equation model was applied for statistical analysis. Results: The relationship between parental obesity and cardiometabolic risk was observed in children and adolescents with adequate sleep, being significant (*p* < 0.05) mediated by physical activity, natural food, physical fitness, and lifestyle. This was also observed when parental education was included in the inadequate sleep cluster model. Conclusion: Present findings underscore the importance of sufficient sleep duration as a critical factor in understanding the complex interplay between parental obesity and cardiometabolic risk in children and adolescents.

## Introduction

Sleep quality is an important component of overall health in all ages, especially in children and adolescents, where it is essential for physical growth, cognitive development, well-being, mental health, and the optimal functioning of various body systems [1, 2]. Insufficient sleep has been associated with poor physical health [3, 4], poor mental health, and problems with attention, behavior, learning, and memory [5–8]. Insufficient sleep includes poor sleep quality and short sleep duration. Sleep deprivation has become common nowadays, as children and adolescents generally sleep less now compared with some decades ago, which is related to changes in lifestyle of modern societies, such as late-night screen time, artificial light, and no bedtime rules at home [9]. Also, long sleep durations can also be associated with health impairments [10]. Therefore, inadequate sleep, characterized by sleeping less or more than recommended, is associated with an increased risk of metabolic and cardiovascular complications, such as obesity, diabetes, and hypertension [10, 11], which can have long-term health consequences in modern-day youth [12].

Inadequate sleep can lead to hormonal imbalances, chronic inflammation, insulin resistance, and impaired glucose metabolism. These factors, in turn, increase the risk of developing cardiometabolic complications [13]. Furthermore, poor sleep quality and chronic sleep deprivation have also been associated with changes in appetite, dysregulation of energy metabolism, weight gain, and resistance to weight loss, which contribute to cardiometabolic risk [14]. A previous study carried out with Spanish children aged 8 to 11 years demonstrated that sub-optimal sleep duration served as a risk factor for cardiometabolic risk [15]. Conversely, in Canadian children and adolescents, short sleep duration was primarily associated with overweight and obesity, but no associations was observed with insulin, cholesterol, and triglyceride levels [16]. Thus, the influence of sleep duration on cardiometabolic risk factors on youth population has been previously discussed, but there are discrepancies through the literature [17]. Disparities could be attributed to several factors that may influence these variables, including lifestyle considerations such as physical activity (PA), dietary habits, body composition, physical fitness, or parental obesity [2, 18–20].

Sleep quality, although it has not been considered in the present study, is an important factor alongside sleep quantity when considering the health impacts on children and adolescents. Sleep quality can be markedly compromised by the presence of sleep-disordered breathing, and obstructive sleep apnea [21, 22]. The presence of obstructive sleep apnea in pediatric populations has been associated with an increased risk of cardiometabolic abnormalities, including hypertension, insulin resistance, dyslipidemia, and obesity [22]. Poor sleep quality has also been associated with symptoms of anxiety and depression among children and adolescents [23].

In the proposed model for this study, we hypothesized that parental obesity plays an important role in cardiometabolic risk factors, depending on the category of sleep duration, and may be influenced by several health indicators. This hypothesis was supported by previous studies suggesting that parents with obesity is linked to higher body mass index (BMI) in their children and an increased risk of cardiometabolic problems [18, 24]. Also, it has been observed that children of obese parents tend to adopt unhealthy habits, including increased sedentary time, low physical activity and unhealthy eating behavior [25, 26]. In addition, parents play a substantial role in shaping the dietary choices of adolescents. Heightened parental stress could potentially impact parenting practices, contributing to an elevated likelihood of stress-induced eating habits and adolescent obesity [27]. Consequently, these factors may contribute to lower physical fitness levels and an increased cardiometabolic risk [28].

By considering these multifaceted factors, we aim to elucidate the intricate pathways through which sleep duration may interact with parental obesity to impact cardiometabolic health outcomes. The independent association between parental obesity, sleep and lifestyle habits with cardiometabolic risk factors in children and adolescents have been widely explored in the literature. Our study represents a novel approach, using a structural equation model, to comprehensively investigate a complex model encompassing various aspects associated with cardiometabolic risk in youth. The structural equation model allows the determination of complex relationships between observed and latent variables, providing insights into relation effects and the overall fit of the theoretical model to empirical data [29]. Therefore, the present study aimed to verify the relationship between parental obesity and cardiometabolic risk in children and adolescents, considering the mediator role of health indicators according to sleep time.

## Methods

This cross-sectional study was conducted on a sample of 3975 children and adolescents (1793 boys) aged 6 to 17 years old, attending public and private schools in Santa Cruz do Sul located in Southern Brazil. The data used in this study is derived from a cohort, where the initial sample was recruited in 2004. The population density of students from all regions of the city was considered to determine the number of participants included in the research. From a total of fifty schools with 20,380 schoolchildren, twenty-five schools were randomly selected to form a representative sample of the city, encompassing schools from different regions of the municipality. All students from these 25 schools were invited to participate in the cohort study, which was divided into multiple phases: Phase I (2004–2005), Phase II (2007–2009), Phase III (2011–2012), Phase IV (2014–2015), and Phase V (2016–2017). For the present study, data from Phase IV and V were considered.

The study was managed in accordance with Resolution 466/2012 of the National Health Council of Brazil, follows the guidelines of Helsinki declaration [30], and received approval from the ethics committee of University of Santa Cruz do Sul (Approval No. 4,278,679). Prior to participation, the parents or legal guardians of the schoolchildren provided signed consent forms.

### Variables

Evaluations took place from 2014 to 2017 in the facilities of the University of Santa Cruz do Sul, conducted by a team of trained researchers. For assessments involving questionnaires, parents provided assistance for children under the age of 10 in answering the questions.

### Sleep time

Sleep time was evaluated through the following questions: “What time do you go to sleep during the week and the weekend?” and “What time do you get up during week and weekend?. These questions were used in previously published paper [31]. To calculate the total sleep time, the questions referring to the week and the weekend were averaged. Sleep time classification was performed according to National Sleep Foundation’s reference values for short time, adequate time and long sleep time (≤ 8 h, 9–11 h and ≥ 12 h for individuals 6–13 years, and ≤ 7 h, 8–10 h and ≥ 11 h for teenagers 14–17 years old, respectively) [32], being recategorized in two categories, adequate and inadequate sleep time (short and long sleep).

### Adiposity indicators

Weight and height measurements were obtained using an anthropometric scale equipped with a stadiometer (Filizola^®^). BMI was calculated by dividing the weight (kilograms) by the square of the height (meters). The body fat percentage was evaluated through the measures of tricipital and subscapular folds, evaluated using a Lange^®^ capilar (Beta Technology Inc, Houston, TX) and then applying the Slaughter et al.’s Eq. [33]. In addition, the obesity information of the parents was obtained through a self-reported questionnaire, where both the mother and father were asked to indicate their obesity status (presence or absence). Information regarding the perception of parents obesity was obtained by a self-reported questionnaire, in which there was a table where mothers/fathers could indicate the presence of cardiac, pulmonary, or circulatory diseases before the age of 55 years. Obesity was one of the included diseases. The questionnaire was sent via the children, to be answered by their parents. The possible answers were yes and no.

### Lifestyle

A self-reported questionnaire was used to evaluate the lifestyle, encompassing five components related to individual well-being [34]: nutrition, PA, preventive behavior, relationships, and stress control. Nutrition included the following questions: (a) Your daily diet includes at least 5 servings of fruits and vegetables; (b) You avoid eating fatty foods (fatty meat, fried foods) and sweets; (c) You eat 4 to 5 varied meals a day, including a full breakfast. Physical activity considered the following questions: (a) You perform at least 30 min of moderate/intense physical activity, continuously or cumulatively, 5 or more days a week; (b) At least twice a week, you perform exercises involving strength and muscle stretching; (c) In your day-to-day life, do you walk or cycle as a means of transport and, preferably, use the stairs instead of the elevator. Preventive behavior was evaluated by questions: (a) You know your blood pressure, your cholesterol levels and try to control them; (b) You do not smoke and do not drink alcohol (or drink in moderation); (c) You respect traffic rules (as a pedestrian, cyclist or driver), if you drive, always wear your seat belt and never drink alcohol. Relationships included the questions: (a) You seek to cultivate friends and is satisfied with your relationships; (b) Your leisure includes meetings with friends, group sports activities, and participation in associations or social entities; (c) You seek to be active in your community, feeling useful in your social environment. Stress control considered the following questions: (a) You set aside time (at least 5 min) every day to relax; (b) You maintain a discussion without changing yourself, even when contradicted; (c) You balance the time dedicated to work with the time dedicated to leisure. All questions had the following options for the responses: never, sometimes, often, and always. The questionnaire was answered by the adolescents, and for children under the age of 10, parents provided assistance in answering the questions.

### Physical activity levels

Physical activity levels were obtained by self-reported questionnaire through the following questions: “Do you usually practice any sport/physical activity?” (yes, or not); “How many times a week and hours/minutes per day do you practice this sport/physical activity”. Thus, the total time (in minutes per week) spent on sports or physical activity was calculated by summing the responses. This value represents the individual’s physical activity levels per week.

### Natural food consumption

To evaluate natural food consumption, the frequency of food intake in a typical week was assessed using a self-reported questionnaire. The questionnaire included the consumption of various natural foods such as fruits, natural fruit juice, potatoes, beans and rice, fish, beef, green salads (lettuce or other), and vegetables (tomatoes, carrots, green beans, cauliflower, etc.). Participants were provided with five response options: never, one time a week, two or three times a week, four to six times a week, and daily.

### Physical fitness

Physical fitness was evaluated according to the protocols of *Projeto Esporte Brasil* (PROESP-Br) [35].

### Cardiorespiratory fitness

Cardiorespiratory fitness (CRF) was evaluated using the six-minute walking and running test. The schoolchildren were instructed to complete as many laps as possible, either running or walking, within a six-minute time frame. The test was conducted on an outdoor athletic track, with markings every 10 m to accurately measure the distance covered (meters). The number of laps completed was recorded, and for those who were unable to complete a full lap, the additional distance covered was also noted. CRF was then determined by multiplying the number of laps by the total distance covered (meters) [35].

### Muscle strength

Abdominal strength was evaluated through the sit-up test, which consisted of determining the sit-ups performed during one minute (repetitions). Upper limb strength was evaluated using a measuring tape fixed to the floor (meters). Participants were seated with their legs together, leaning their body against the wall. They were instructed to flex their arms and throw a medicine ball. Two attempts were made, and the longest distance achieved was recorded as the measurement for upper limb strength (meters) [35].

### Agility and speed

Agility was measured using the square test, which involved placing cones at the four corners of a square. The participant started from a designated point and moved towards the diagonally opposite cone, then proceeded to the cone on their left, diagonally to the next cone, and finally moved towards the last cone to complete the test. Participants were required to move at their maximum speed and touch each cone. Speed was evaluated by timing (seconds) the children and adolescents as they ran a distance of 20 m as quickly as possible. The best time recorded from two attempts was used for both the agility and speed assessments [35].

### Cardiometabolic risk indicators

To assess systolic blood pressure (mmHg), the auscultatory method was employed, utilizing a sphygmomanometer and a stethoscope in accordance with the recommendations outlined in the VII Guidelines of the Brazilian Society of Cardiology (2016) and [36]. Two measures were performed after five minutes of rest, and the lowest result was considered for systolic blood pressure. Waist circumference (centimeters) was obtained in the narrowest part of the trunk between the last rib and the iliac crest [37], using an inelastic tape with a resolution of 1 mm (Cardiomed^®^, Brazil).

Triglycerides (mg/dL), total cholesterol (TC) (mg/dL), high-density lipoprotein cholesterol (HDL-C) (mg/dL), and fasting glucose(mg/dL) were evaluated through a blood sample collection after 12 h of fasting. Blood collection was realized by morning and used serum samples and commercial kits (DiaSys Diagnostic Systems, Holzheim, Germany), performed on Miura 200 automated equipment (I.S.E., Rome, Italy).

The clustered metabolic risk score (cMetS) considered the summing z-scores of each factor risk (waist circumference, systolic blood pressure, triglycerides, TC/HDL-C ratio, and fasting glucose) divided by five. To calculate sex and age-specific standardized z-scores were considered according to an international reference [38] for each risk factor with the following equation: z-score = ([X - X̅]/SD); where X is the measured continuous value of the risk factor; X̅ is the predicted mean calculated for the cMetS risk factor using the sex- and age-specific international reference equation; and standard deviation (SD) is the international SD for the specific each risk factor. Before calculating z-scores, TC/HDL-C ratio and triglycerides were log-transformed using the natural logarithm because of their skewness.

### Parental education level

The determination of parental education level consisted of a self-reported question, in which parents should indicate their level of education, according to the following options: Illiterate/Incomplete Elementary Education (up to 3rd Grade), Complete Elementary Education/Incomplete Elementary Education, Complete Middle School/Incomplete High School, Complete High School/Incomplete Higher Education, Complete Higher Education.

### Statistical analysis

**Fig. 1:**
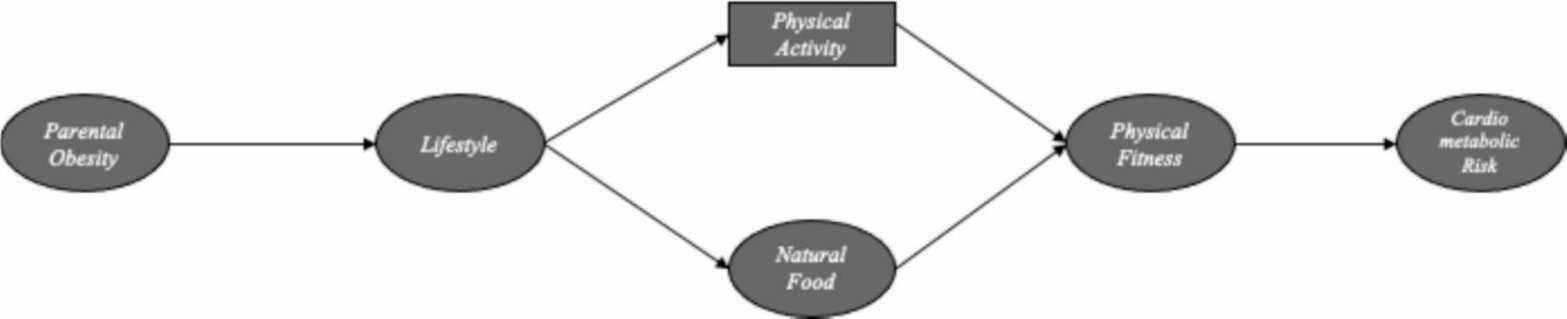
Theoretical model proposal to determine the relationship between parental obesity and cardiometabolic risk factors in children and adolescents, considering the mediator role of health indicators accordingly to sleep time

Based on the objectives of the present study and previous literature [39, 40], we developed a structural equation model (Fig. 1) to examine the complex relationships between parental obesity and cardiometabolic risk factors in children and adolescents with both inadequate and adequate sleep time, considering the aforementioned variables. Within this model, lifestyle, PA levels, natural food consumption, and physical fitness were considered interconnected mediators. The latent constructs presented in Fig. 1 were defined as follows: parental obesity, lifestyle habits (nutrition, PA, preventive behavior, relationships, and stress control); natural food consumption (fruits, natural fruit juice, potatoes, beans and rice, fish, beef, green salads, and vegetables; physical fitness (CRF, abdominal strength, agility, speed, and upper limb strength); Cardiometabolic risk factors (cMetS), and adiposity indicators [BMI, and body fat percentage]).

Based on these primary aspects, the data were evaluated using structural equation models (SEM) with a split file for two classifications based on sleep time (adequate and inadequate). In addition, considering a discrepant of theoretical consistency in primary results to the model for inadequate sleep time children, we input an alternative model for this group, with a correction to the parental education level.

**Fig. 2:**
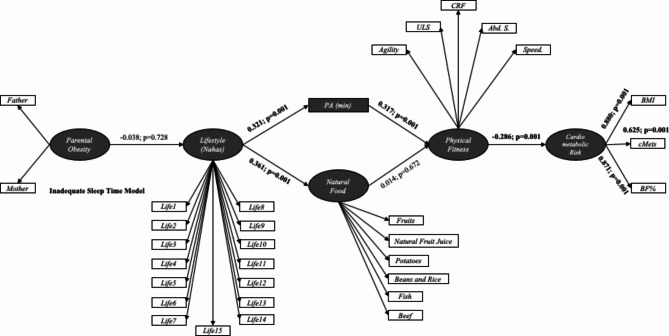
Structural equation models for cluster with inadequate sleep time model Lifestyle (Nahas): Life1-15. Questions regarding lifestyle and quality of life (nutrition, physical activity, preventive behavior, relationships, and stress control); PA. Physical Activity; ULS. Upper limb strenght; CRF. Cardiorespiratory fitness; Abd. S. Abdominal strenght; BMI. Body mass index; cMetS. Clustered metabolic risk score; BF%. Body fat percentage

**Fig. 3:**
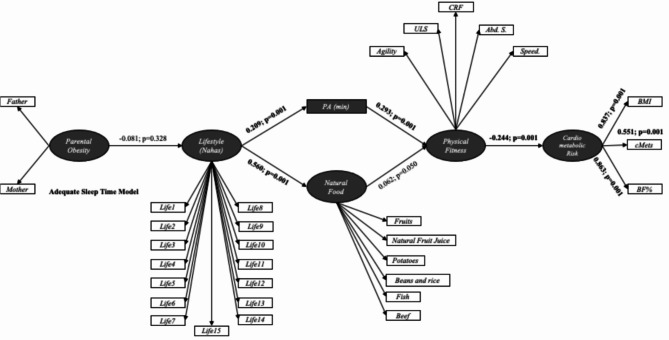
Structural equation models for cluster with adequate sleep time model Life1-15. Questions regarding lifestyle and quality of life (nutrition, physical activity, preventive behavior, relationships, and stress control); PA. Physical Activity; ULS. Upper limb strength; CRF. Cardiorespiratory fitness; Abd. S. Abdominal strength; BMI. Body mass index; cMetS. Clustered metabolic risk score; BF%. Body fat percentage

The direct and indirect effects are estimated based on the beta (β) values of standardized variables, to be possible to measure the factorial load importance of each variable included in the model. The goodness-of-fit criteria used to compare the groups are as follows [29]: CMIN/DF: This criterion measures the ratio of the chi-square statistic (CMIN) to the degrees of freedom (DF). It assesses the disparity between the observed and expected covariance matrices. A lower CMIN/DF value indicates a better fit, with a value close to 1 being desirable. IFI (Incremental Fit Index): IFI compares the proposed model with a null model to measure the improvement in fit. It ranges from 0 to 1, with values closer to 1 indicating a better fit. TLI (Tucker-Lewis Index): TLI also compares the proposed model with a null model and quantifies the degree of improvement in fit. Similar to IFI, TLI ranges from 0 to 1, with values above 0.90 indicating a better fit. FMIN: This criterion represents the minimum discrepancy between the observed and predicted covariance matrices. A lower FMIN value indicates a better fit. RMSEA (Root Mean Square Error of Approximation): RMSEA measures the discrepancy between the predicted model and the population covariance matrix, considering the model’s complexity. It provides an index of how well the model fits the data, with a lower RMSEA value (close to 0) indicating a better fit. Values below 0.05 are often considered good. AIC (Akaike Information Criterion): AIC is a measure of model fit those accounts for model complexity. It balances goodness of fit with model complexity, with lower AIC values indicating a better fit. BIC (Bayesian Information Criterion): Similar to AIC, BIC considers both goodness of fit and model complexity. It penalizes more complex models and provides a measure of relative fit. Lower BIC values indicate a better fit. By applying these criteria, we can determine if the groups with adequate or inadequate sleep time are statistically the most suitable to be represented by the proposed SEM [29] (Figs. 1 and 2, and 3). The statistical program used for the analysis was IBM AMOS version 21.0.

## Results

Table 1 presents the characteristics of the sample according to boys and girls.

**Table 1:** Descriptive sample characteristics

Latent Constructs	Variables included in study	Total (3973)			Boys (1732)			Girls (2243)	
		Mean	SD		Mean	SD		Mean	SD
**Parents**	***Education level of the mother***	3.05	1.27		3.12	1.29		2.99	1.25
	***Education level of the father***	2.84	1.28		2.89	1.27		2.81	1.28
	***Father with obesity***	1.05	0.21		1.05	0.21		1.05	0.21
	***Mother with obesity***	1.08	0.27		1.08	0.26		1.08	0.27
	***Age***	11.61	2.78		11.51	2.83		11.69	2.74
**Fitness**	***Agility***	7.08	1.01		6.77	0.93		7.32	1.00
	***Uper Limbs Strenght***	3.01	2.76		3.32	3.09		2.77	2.44
	***CRF***	881.43	196.47		967.46	221.59		812.54	139.77
	***Abdominal Strenght***	23.87	9.15		27.07	9.73		21.35	7.80
	***Speed***	4.59	0.81		4.35	0,76		4.77	0.80
**Cardiometabolic** **Risk**	***BMI***	20.39	4.30		20.19	4.15		20.55	4.40
	***Skinfolds Sum***	28.26	13.76		24.57	13.06		31.17	13.60
	***Total Cholesterol***	160.92	31.63		157.72	31.64		163.44	31.39
	***Glucose***	88.89	16.01		89.74	9.15		88.22	19.77
	***HDL-C***	59.14	11.35		59.94	11.72		58.50	11.01
	***LDL-C***	87.25	27.24		84.53	26.65		89.38	27.51
	***TGO***	20.95	7.92		22.07	7.22		20.07	8.34
	***TGP***	17.15	10.88		17.65	8.27		16.77	12.54
	***Triglicerides***	72.76	140.21		64.60	31.44		79.16	185.03
	***cMets***	-0.07	0.70		-0.13	0.70		-0.02	0.69
	***Minutes of physical Activity a week***	122.36	187.90		154.30	204.06		98.07	170.70
**Lifestyle**	***Does your daily diet include at least 5 servings of fruits and vegetables?***	2.10	0.77		2.12	0.77		2.09	0.77
	***Do you avoid consuming fatty foods (fatty meats***,*** fried foods) and sweets?***	2.15	0.97		2.16	0.83		2.14	1.06
	***Do you have 4 to 5 varied meals a day***,*** including a complete breakfast?***	2.80	1.56		2.80	1.13		2.79	1.83
	***Do you engage in at least 30 min of moderate/intense physical activity***,*** either continuously or accumulated***,*** 5 or more days a week?***	2.42	1.72		2.67	1.98		2.22	1.46
	***Do you perform strength and stretching exercises at least 2 times a week?***	2.52	1.46		2.69	1.62		2.38	1.31
	***In your daily life***,*** do you walk or cycle as a means of transportation and preferably use stairs instead of the elevator?***	2.62	1.47		2.66	1.16		2.59	1.68
	***Do you know your blood pressure and cholesterol levels and seek to control them?***	1.75	1.32		1.77	1.25		1.73	1.38
	***Do you not smoke and do not consume alcohol (or consume it in moderation)?***	3.34	1.74		3.32	1.80		3.35	1.69
	***Do you respect traffic regulations (as a pedestrian***,*** cyclist***,*** or driver)***,*** always wear your seatbelt when driving***,*** and never consume alcohol?***	3.55	1.27		3.50	1.33		3.59	1.21
	***Do you seek to cultivate friendships and are satisfied with your relationships?***	3.61	1.56		3.62	1.70		3.60	1.44
	***Does your leisure time include gatherings with friends***,*** group sports activities***,*** or participation in associations or social organizations?***	2.99	1.27		3.05	1.22		2.93	1.31
	***Do you seek to be active in your community***,*** feeling useful in your social environment?***	2.82	1.51		2.85	1.46		2.80	1.55
	***Do you set aside time (at least 5 min) every day to relax?***	2.97	1.18		2.99	1.27		2.94	1.10
	***Do you maintain a discussion without getting upset***,*** even when contradicted?***	2.26	0.97		2.33	0.96		2.21	0.98
	***Do you balance the time devoted to work with leisure time?***	2.75	1.08		2.75	1.05		2.74	1.11
**Nutrition**	***How often do you eat green salads (lettuce or other) and vegetables (tomatoes***,*** carrots***,*** green beans***,*** cauliflower***,*** etc.)?***	3.00	2.37		2.90	2.45		3.07	2.31
	***How often do you eat beef?***	3.45	1.30		3.51	1.25		3.40	1.34
	***How often do you eat fish?***	1.22	1.18		1.29	1.21		1.16	1.16
	***How often do you drink natural fruit juice? (excluding soft drinks or artificial beverages)***	2.36	1.45		2.40	1.47		2.33	1.44
	***How often do you eat fruits (excluding fruit juice)?***	3.20	1.48		3.24	1.64		3.17	1.35
	***How often do you eat potatoes (excluding french fries or chips)?***	2.42	1.82		2.42	1.82		2.43	1.82
	***How often do you eat beans with rice?***	4.19	1.55		4.25	1.63		4.13	1.48

Table 2 displays the goodness-of-fit results for the two clusters of sleep time analyzed in the SEM. Based on these findings, we can confidently state that the SEM is statistically well-suited to represent the data concerning children and adolescents with sufficient sleep time. In other words, the multiple relationships presented in this study exhibit a higher degree of consistency within this group compared to the cluster characterized by inadequate sleep time, and this was also observed in the alternative model. This is concluded based on the following parameters: the lower the CMIN/DF values, the better the models are. Also, a better model fit is observed when IFI, TLI, and CMIN values are closer to 0.99. The RMSEA should be below or close to 0.08. Finally, AIC and BIC should have the lowest possible values.

**Table 2:** Parameters of adjustment for the three structural models according to sleep time

SEM Parameters	Inadequate Sleep Time	Adequate Sleep Time	Alternative model Inadequate Sleep Time
CMIN/DF	5.52	4.74	5.31
IFI	0.74	0.79	0.76
TLI	0.72	0.77	0.74
FMIN	1.52	0.99	1.66
RMSEA	0.05	0.04	0.049
AIC	2913	2532	3170
BIC	2917	2535	3174

SEM. Structural equation models; CMIN. Criterion measures the ratio of the chi-square statistic; DF. Degrees of freedom; IFI. Incremental Fit Index; TLI. Tucker-Lewis Index; FMIN. Criterion represents the minimum discrepancy between the observed and predicted covariance matrices; RMSEA. Root means square error of approximation; AIC. Akaike information criterion; BIC. Bayesian information criterion

Figure 2 presents the primary direct relationships among the variables included in the proposed SEM applied to the cluster with inadequate sleep time. The variable “Lifestyle” exhibits a positive association with both PA (*p* = 0.001) and natural food consumption (*p* = 0.001). PA, in turn, demonstrates a positive association with physical fitness (*p* = 0.001), indicating that being more physically active correlates with improved fitness levels in this group. Furthermore, physical fitness exerts a negative influence on cardiometabolic risk (*p* = 0.001), suggesting that higher levels of physical fitness are associated with reduced risk factors. The construct of cardiometabolic risk displays strong correlations (β > 0.600) with all observable indicators, namely BMI, cMetS, and body fat percentage. According to this SEM, it can be suggested that parent’s obesity does not have a direct association with lifestyle. Additionally, there is no relationship observed between natural food consumption and the physical fitness construct.

Figure 3 illustrates SEM for the cluster with Adequate Sleep time. These results demonstrate similarities with the SEM depicted in Fig. 2 in terms of significant relationships and their directionality. However, the strength of associations differs between the two models. Specifically, the associations between lifestyle and PA, PA and physical fitness, physical fitness and the cardiometabolic risk construct, as well as the associations of the cardiometabolic risk construct with BMI, cMetS, and percentage of body fat are weaker in the model for the cluster with adequate sleep time compared to the model for the cluster with inadequate sleep time. Conversely, the association between lifestyle and natural food consumption is stronger in children and adolescents with adequate sleep time.

Table 3 presents the mediated (indirect) associations between all variable domains included in the SEM model for the two clusters of sleep time. It is important to note that there are indirect associations between lifestyle and physical fitness, which are slightly stronger in the cluster with inadequate sleep time. These associations are mediated by variables such as PA and natural food consumption. In both clusters, lifestyle, PA, and physical fitness are negatively and indirect associated with the cardiometabolic risk construct and its indicators. However, these associations are stronger in the cluster with inadequate sleep time for children and adolescents.

**Fig. 4:**
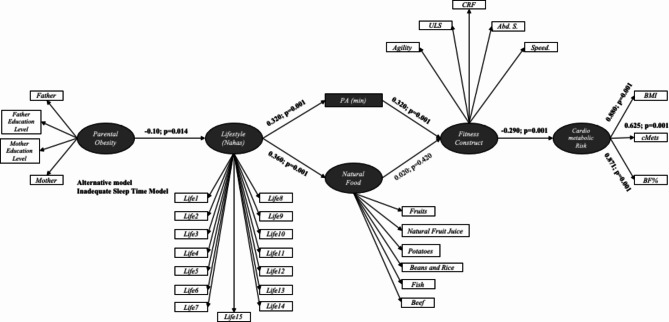
Structural equation models for cluster with inadequate sleep time at alternative model with parents education levelLife1-15. Questions regarding lifestyle and quality of life (nutrition, physical activity, preventive behavior, relationships, and stress control); PA. Physical Activity; ULS. Upper limb strength; CRF. Cardiorespiratory fitness; Abd. S. Abdominal strenght; BMI. Body mass index; cMetS. Clustered metabolic risk score; BF%. Body fat percentage

In the cluster with adequate sleep time, parental obesity only shows negative, mediated associations with PA, natural food consumption, and physical fitness. It exhibits a positive correlation with the cardiometabolic risk construct. These relationships do not occur in the cluster with inadequate sleep time (Table 4; Fig. 4).

**Table 3:** Indirect relationships (mediated) between variables in the models according to inadequate and adequate children and adolescents sleep time

	Inadequate Sleep Time											Adequate Sleep Time									
Independent	Parental Obesity		Lifestyle		Physical Activity		Natural Food		Physical Fitness			Parental Obesity		Lifestyle		Physical Activity		Natural Food		Fitness Construct	
Dependent	β	*p*	β	*p*	β	*p*	β	*p*	β	*p*		β	*p*	β	*p*	β	*p*	β	*p*	β	*p*
PA (minutes)	-0.012	0.062										-0.017	0.015								
Speed (seconds)	0.003	0.062	-0.070	0.001	-0.207	0.001	-0.009	0.868				0.005	0.015	-0.067	0.001	-0.204	0.001	-0.043	0.072		
Upper limb strength (cm)	-0.001	0.062	0.023	0.001	0.069	0.001	0.003	0.868				-0.001	0.015	0.019	0.001	0.057	0.001	0.012	0.072		
Abdominal strength (cm)	-0.003	0.062	0.074	0.001	0.220	0.001	0.010	0.868				-0.005	0.015	0.065	0.001	0.200	0.001	0.042	0.072		
CRF (m)	-0.003	0.062	0.073	0.001	0.215	0.001	0.010	0.868				-0.005	0.015	0.065	0.001	0.200	0.001	0.042	0.072		
Agility (seconds)	0.003	0.062	-0.076	0.001	-0.224	0.001	-0.010	0.868				0.005	0.015	-0.063	0.001	-0.192	0.001	-0.040	0.072		
Fitness construct	-0.004	0.062	0.107	0.001								-0.008	0.015	0.096	0.001						
Body fat percentage	0.001	0.062	-0.027	0.001	-0.079	0.001	-0.004	0.868	-0.249	0.001		0.002	0.015	-0.020	0.001	-0.061	0.001	-0.013	0.072	-0.210	0.001
BMI (kg/m2)	0.001	0.062	-0.027	0.001	-0.080	0.001	-0.004	0.868	-0.252	0.001		0.002	0.015	-0.019	0.001	-0.060	0.001	-0.013	0.072	-0.204	0.001
Cardiometabolic risk	0.001	0.062	**-0.031**	**0.001**	**-0.091**	**0.001**	-0.004	0.868				**0.002**	**0.015**	**-0.023**	**0.001**	**-0.071**	**0.001**	-0.015	0.072		
cMetS	0.001	0.062	-0.019	0.001	-0.057	0.001	-0.003	0.868	-0.179	0.001		0.001	0.015	-0.013	0.001	-0.039	0.001	-0.008	0.072	-0.134	0.001
Healthy Food	-0.014	0.062										-0.045	0.015								
Fruits	-0.009	0.062	0.223	0.001								-0.022	0.015	0.270	0.001						
Natural fruit juice	-0.006	0.062	0.157	0.001								-0.022	0.015	0.275	0.001						
Potatoes	-0.001	0.063	0.037	0.001								-0.009	0.015	0.116	0.001						
Beans and rice	-0.002	0.062	0.047	0.001								-0.009	0.015	0.105	0.001						
Fish	-0.003	0.062	0.091	0.001								-0.015	0.015	0.191	0.001						
Beef	-0.002	0.062	0.051	0.001								-0.009	0.015	0.109	0.001						
Green salads and vegetables	-0.007	0.062	0.182	0.001								-0.014	0.015	0.175	0.001						
cMetS	0.001	0.062	-0.019	0.001	-0.057	0.001	-0.003	0.868	-0.179	0.001		0.001	0.015	-0.013	0.001	-0.039	0.001	-0.008	0.072	-0.134	0.001
Stress 1	-0.010	0.062										-0.027	0.015								
Stress 2	-0.009	0.062										-0.023	0.015								
Stress 3	-0.010	0.062										-0.033	0.015								
Relationship 1	-0.007	0.062										-0.010	0.015								
Relationship 2	-0.013	0.062										-0.028	0.015								
Relationship 3	-0.011	0.062										-0.026	0.015								
Behavior 1	-0.014	0.062										-0.016	0.015								
Behavior 2	0.000	0.969										-0.002	0.382								
Behavior 3	-0.004	0.072										-0.014	0.015								
PA 1	-0.024	0.062										-0.023	0.015								
PA 2	-0.022	0.063										-0.024	0.015								
PA 3	-0.009	0.062										-0.016	0.015								
Nutrition 1	-0.010	0.062										-0.030	0.015								
Nutrition 2	-0.007	0.062										-0.015	0.015								
Nutrition 3	-0.009	0.062										-0.016	0.015								

PA. Physical activity; CRF. Cardiorespiratory fitness; cMetS. Clustered cardiometabolic risk score; BMI. Body mass index

Stress1. Set aside time (at least 5 min) every day to relax; Stress2. Maintain a discussion without changing yourself, even when contradicted; Stress3. Balance the time dedicated to work with the time dedicated to leisure; Relationship (1) Seek to cultivate friends and are satisfied with your relationships; Relationship (2) Leisure includes meetings with friends, group sports activities, participation in associations or social entities; Relationship 3; Seek to be active in your community, feeling useful in your social environment; Behavior (1) Know your blood pressure, your cholesterol levels and try to control them; Behavior (2) Do not smoke and do not drink alcohol (or drink in moderation); Behavior (3) Respect traffic rules (as a pedestrian, cyclist or driver), if you drive, always wear your seat belt and never drink alcohol; PA (1) At least 30 min of moderate/intense physical activity, continuously or cumulatively, 5 or more days a week; PA (2) Exercises involving strength and muscle stretching at least twice a week; PA (3) Walk or cycle as a means of transport; Nutrition (1) Daily diet includes at least 5 servings of fruits and vegetables; Nutrition (2) Avoid eating fatty foods and sweets; Nutrition (3) Eat 4 to 5 varied meals a day, including a full breakfast

Table 4 presents the indirect effects in children and adolescent’s alternative inadequate sleep time model with inclusion of parent’s instruction in the SEM.

**Table 4:** Alternative model for inadequate sleep time indirect relationships considering parental education level

Independent	Parental Obesity		Lifestyle		PA (time)		Natural Food		Fitness Construct	
Dependent	β	*p*	β	*p*	β	*p*	β	*p*	β	*p*
Physical activity	0.032	0.001								
Physical Fitness	0.011	0.001	0.107	0.001						
Cardiometabolic risk	-0.003	0.001	-0.031	0.001	-0.091	0.001	-0.004	0.851		
Healthy Food	0.036	0.001								
Fruits	0.022	0.001	0.223	0.001						
Natural fruit Juice	0.016	0.001	0.156	0.001						
Potatoes	0.004	0.001	0.037	0.001						
Beans and rice	0.005	0.001	0.046	0.001						
Fish	0.009	0.001	0.091	0.001						
Beef	0.005	0.001	0.051	0.001						
Green salads and vegetables	0.018	0.001	0.182	0.001						
Speed	-0.007	0.001	-0.070	0.001	-0.207	0.001	-0.009	0.851		
Upper limb strength	0.002	0.001	0.023	0.001	0.069	0.001	0.003	0.851		
Abdominal strength	0.007	0.001	0.075	0.001	0.220	0.001	0.010	0.851		
CRF	0.007	0.001	0.073	0.001	0.215	0.001	0.010	0.851		
Agility	-0.008	0.001	-0.076	0.001	-0.224	0.001	-0.010	0.851		
cMets	-0.002	0.001	-0.019	0.001	-0.057	0.001	-0.003	0.851	-0.179	0.001
Body fat Percentage	-0.003	0.001	-0.027	0.001	-0.079	0.001	-0.004	0.851	-0.249	0.001
BMI	-0.003	0.001	-0.027	0.001	-0.080	0.001	-0.004	0.851	-0.252	0.001
Stress 1	0.027	0.001								
Stress 2	0.023	0.001								
Stress 3	0.026	0.001								
Relationship 1	0.020	0.001								
Relationship 2	0.035	0.001								
Relationship 3	0.030	0.001								
Behavior 1	0.038	0.001								
Behavior 2	0.001	0.922								
Behavior 3	0.012	0.010								
PA 1	0.061	0.001								
PA 2	0.056	0.001								
PA 3	0.025	0.001								
Nutrition 1	0.026	0.001								
Nutrition 2	0.019	0.001								
Nutrition 3	0.024	0.001								

PA. Physical activity; CRF. Cardiorespiratory fitness; cMetS. Cardiodetabolic risk score; BMI. Body mass index;

Stress1. Set aside time (at least 5 min) every day to relax; Stress 2. Maintain a discussion without changing yourself, even when contradicted; Stress 3. Balance the time dedicated to work with the time dedicated to leisure; Relationship (1) Seek to cultivate friends and are satisfied with your relationships; Relationship (2) Leisure includes meetings with friends, group sports activities, participation in associations or social entities; Relationship 3; Seek to be active in your community, feeling useful in your social environment; Behavior (3) Respect traffic rules (as a pedestrian, cyclist or driver), if you drive, always wear your seat belt and never drink alcohol; Behavior 2. Do not smoke and do not drink alcohol (or drink in moderation); Behavior (1) Know your blood pressure, your cholesterol levels and try to control them; PA 3. Walk or cycle as a means of transport; PA (2) Exercises involving strength and muscle stretching at least twice a week; PA 1. At least 30 min of moderate/intense physical activity, continuously or cumulatively, 5 or more days a week; Nutrition (1) Daily diet includes at least 5 servings of fruits and vegetables; Nutrition (2) Avoid eating fatty foods and sweets; Nutrition (3) Eat 4 to 5 varied meals a day, including a full breakfast

## Discussion

The main findings of the present study revealed that the relationship between parental obesity and cardiometabolic risk was observed in children and adolescents with adequate sleep, being significantly mediated by PA, natural food, physical fitness, and lifestyle. This also held true when we included the parental education factor in the inadequate sleep cluster model. These results emphasize the importance of sufficient sleep time in the connection between the studied variables and their impact on cardiometabolic risk. Additionally, findings underscore the negative influence of parental obesity associated with low family educational level, considering this has a detrimental effect on cardiometabolic health, even when accounting for lifestyle factors, among others. This effect is particularly pronounced when analyzing the inadequate sleep time group. Our study suggests that the proposed model can consistently identify associations when applied to data of children with adequate sleep, whereas the same model did not hold mathematically for children and adolescents with inadequate sleep. This implies that, within the same population, inadequate sleep significantly affects several aspects of the health of children and adolescents.

It is known that inadequate sleep time (short and long) presents negative effects on health in pediatric population [10, 41]. In this sense, it was observed that short sleep (less than six hours) plays a key role in obesity, mortality, diabetes, coronary heart disease, and cardiovascular disease [4]. Another study also verified that short sleep time is associated with increased obesity central and total, while long sleep is related to elevated triglycerides [42]. The sleep hours alteration is usually associated with the use of smartphone before bedtime, which negatively influences daily functioning and mood [43]. Other factors can also interfere with sleep time, like an increase in age, study shift, and time spent in sedentary behavior [44].

In addition, parental lifestyle negatively influences the lifestyle of obese children [18, 45] especially when individuals are living in vulnerable situations with limited access to education [39, 40, 46–48], which corroborates current findings. Children and adolescents with overweight have higher odds of having a father with hypertension and a mother with obesity [45]. In addition, children that have parents with hypertension present higher abdominal obesity, blood pressure and alanine transaminase [49]. Hence, this underscores the significance of ensuring that the younger population engages in regular PA, as it proves advantageous for health markers. Notably, increased levels of CRF and muscular strength appear to mitigate the adverse impact of maternal obesity on elevated BMI [18]. PA levels are lower in obese children with more [30] sedentary parents [50]. Evidence also suggests that the adoption of a healthy lifestyle by parents risks reduced obesity in children and adolescents [51–53].

Our hypothesis about the findings of the present study is that in the adequate sleep time group, the children and adolescents present better health indicators, like a healthy lifestyle and high physical fitness levels. It was observed that in the adequate sleep time group, there was a stronger relationship between lifestyle and natural food consumption compared with the inadequate sleep time group. From this, it can be suggested that in this group, parental obesity does not directly influence the lifestyle of their children. However, for children and adolescents that present lower PA, natural foods consumption and physical fitness and only adequate sleep time, parental obesity is positively associated with cardiometabolic risk factors. Differently observed in inadequate sleep time that demonstrates that parental obesity is associated with children’s lifestyle. However, it is important to reiterate that there is not enough theoretical consistency to interpret that only parental obesity has relationships mediated by PA, natural food, physical fitness, and lifestyle in the context of cardiovascular risk in this study, especially when we do not take into account the education level of parents.

Indeed, sleep times greatly influence the behavior and overall health of children and adolescents. Therefore, complying with sleep recommendations is very important in several health contexts once the odds of developing cardiometabolic complications are lower in the adequate sleep time group [42]. From this, intervention proposals regarding lifestyle habits should primarily take into account sleep duration, as children’s behavior varies and has an impact on health. In this sense, it is highlighted the importance of developing actions targeting sleep health, like encouraging the adoption of active behavior, the practice of PA, and the reduction of sedentary behavior [10]. In addition, incorporating lectures that emphasize the essential role of sleep in facilitating daily tasks, aiming to enhance awareness of its importance, targeting both the students and their families [54]. Parental influences was also evident in behavioral choices related to PA and screen time [53].

Our study is not without limitations. Sleep time was assessed using self-reported questionnaires, which offer a cost-effective and efficient means to gather information on children’s sleep hygiene. However, it is important to acknowledge the inherent limitations of questionnaire-based assessments. These methods rely on subjective self-reporting, which may introduce biases and inaccuracies, especially among younger children or those with developmental or cognitive impairments. Furthermore, our study did not account for napping or daytime sleep, and it did not assess sleep quality or the presence of sleep-disordered breathing, which can be more accurately evaluated using polysomnography, recognized as the gold standard for diagnosing sleep disorders in children [21]. Polysomnography provides comprehensive physiological data during sleep, including brain activity, muscle movements, and respiratory patterns, although its cost and duration may limit its accessibility [21]. Also, assessing parental obesity, physical activity and food consumption using self-reported questionnaires may potentially result in underestimation or overestimation of activity levels or food intake. The use of questions to ascertain parental obesity, while convenient due to our large sample size, lacks the depth and accuracy that more comprehensive assessments would provide. Thus, our study´s findings should be interpreted within this context. Finally, caution is warranted in interpreting the results due to the cross-sectional design of the study, which does not permit the confirmation of causal relationships.

The present study also presents some strengths that must be highlighted. The study approaches consistent data from a representative sample of the population and fills a gap in the literature related to understanding the complexity of the relationship between parental obesity, sleep, and behavioral and physiological health. To the best of our knowledge, this is the first study that attempts to integrate and examine the interplay between sleep time, parental obesity, and other lifestyle habits within a unified framework. By doing so, we can better understand the synergistic effects and potential mediating mechanisms that underlie the relationship between these factors and cardiometabolic health in young individuals. In other words, it is not possible to simplify the same idea of relationships between multiple variables without considering the influence of these variables on the overall behavior of others at the time of assessment. Our research seeks to address important research gaps and provide a comprehensive analysis of these interconnected factors, thereby contributing to the existing body of knowledge and paving the way for targeted interventions and preventive strategies to mitigate cardiometabolic risk in youth. Furthermore, our study represents one of the pioneering efforts in Latin America to assess health lifestyles, cardiometabolic risk, and physical education variables using a complex structural equation model. These research methodologies, while more established in countries like India, still remain relatively novel in South Americans countries such as Brazil.

Taking all our results into account, in addition to the currently literature available, sleep time in childhood and adolescence is a public health issue, and there is a clear need for sleep restriction/extension interventions, as well to determine upper and lower limits of healthy sleep duration, to understand a dose–response curve for different ages [17]. For clinical practice, the effectiveness of different interventions to improve sleep in short/long sleepers on reducing cardiometabolic risks should be examined on large scale and in the general community settings.

## Conclusions

Our study revealed that the relationship between parental obesity and cardiometabolic risk in children and adolescents is influenced by various health indicators, particularly in those with adequate sleep. Factors such as PA, natural food consumption, physical fitness, and lifestyle play significant mediating roles in this relationship. Additionally, when we consider parental education, especially in the context of inadequate sleep, the negative influence of parental obesity on cardiometabolic health becomes more pronounced. These findings underscore the importance of sufficient sleep time as a critical factor in understanding the complex interplay between these variables. Moreover, it’s noteworthy that the structural equation models applied to the cluster with inadequate sleep time demonstrated less consistency in terms of fit. In practice, this implies that this group exhibits less behavioral homogeneity, presenting a challenge for future research to identify the factors influencing these relationships. This underscores the need for more in-depth investigations to unravel the intricacies of cardiometabolic risk in children and adolescents, especially those facing inadequate sleep patterns.

## Data Availability

The datasets used and/or analysed during the current study are available from the corresponding author on reasonable request.

## Abbreviations

PA: Physical activity
BMI: Body mass index
CRF: Cardiorespiratory fitness
TC: Total cholesterol
HDL-C: High-density lipoprotein cholesterol
SD: Standard deviation
cMetS: Cardiometabolic risk factors
SEM: Structural equation models
CMIN: Ratio of the chi-square statistic
DF: Degrees of freedom
IFI: Incremental Fit Index
TLI: Tucker-Lewis Index
RMSEA: Root Mean Square Error of Approximation
